# THE IMPORTANCE OF UNDERSTANDING KINSHIP STRUCTURES FOR LEFT BEHIND OLDER ADULTS IN HIGH OUTMIGRATION CONTEXTS

**DOI:** 10.1093/geroni/igae098.1024

**Published:** 2024-12-31

**Authors:** Amilcar Matos-Moreno

**Affiliations:** The Pennsylvania State University, San Juan, Puerto Rico

## Abstract

From early in my scientific career (doctoral student), the National Institute on Aging and the Gerontological Society of America have been a cornerstone of developing my scientific understanding, from training grants that allowed me to finish my Ph.D. to grant writing workshops that helped me land a K01 award. Participating in the RAND Summer Institute and the Butler Williams Scholar program gave me the tools to understand the many funding mechanisms within the NIA and clarify the many doubts that come with transitioning into a faculty position. Further, my research has evolved in ways that I could never imagine. My studies surround rapid aging and migration in Puerto Rico and other countries. I study the intertwined pathways between migration, left behind older adults, and kinlessness in the 21st century. Specifically, how the migration of working-age adults is increasing the number of kinless older adults in sending countries and the effect of reducing social networks on the health and well-being of left behind older adults. Furthermore, our current work projects the kinship structure of older adults in Puerto Rico and Mexico, considering different migratory scenarios.

